# Editorial: Aquatic photosynthetic organisms under global change

**DOI:** 10.3389/fpls.2024.1539716

**Published:** 2025-01-23

**Authors:** Justine Marchand, Benoît Schoefs

**Affiliations:** Metabolism, Molecular Engineering of Microalgae and Applications, Laboratory Biology of Organisms, Stress, Health and Environment, IUML – FR 3473 CNRS, Le Mans University, Le Mans, France

**Keywords:** adaptation, algae, carbon metabolism, freshwater, global warming, photosynthesis, plant, ocean

From space, Earth looks like a masterful stone marquetry such as can be admired in Florence, Italy. In this stone painting, large areas and thinner lines of lapis lazuli would represent oceans (97%) and rivers (3%), respectively. Together, they occupy 71% of the Earth’s surface. Despite their different chemical composition, oceans and rivers host a large number of living organisms, including phototrophs. Their diversity is also great with anoxygenic and oxygenic bacteria, and unicellular and multicellular eukaryotes, including some flowering plants. For example, the number of identified alga species lies in the range of 40–60,000 (Guiry, 2024), a number that increases annually (e.g., Schoefs et al., 2020; Morin et al., 2025). As primary producers, aquatic photosynthetic organisms fulfill crucial ecosystem services for the planet. This includes approximately half of the global oxygen production (Benoiston et al., 2017) and photosynthetic carbon fixation (Falkowski and Raven, 2013). Aquatic photosynthetic organisms are also involved in many spectacular symbioses with non-photosynthetic organisms such as the symbiosis between the ciliate *Paramecium bursaria* and the green freshwater microalga *Chlorella variabilis* (Kodama and Sumita, 2022), allowing the partners to develop during dedicated phase or accompanying them all along their life like the symbiotic dinoflagellates in corals. In addition to the obvious freshwater, brackish water, and saline water habitats, aquatic photosynthetic organisms are also present in more surprising and/or drastic niches such as solid water (e.g., Procházková et al., 2024), hot springs (Smith et al., 2013), radioactive natural springs (Millan et al., 2020), and loggerhead sea turtles (Majewska et al., 2020). Each of these aquatic organisms develops according to its ecological optimum. When one ecological factor deviates from its optimum, aquatic photosynthetic organisms start to acclimate.

Global warming has direct and indirect impacts on the climate and therefore on the ecological parameters of every ecological niche, including aquatic ones. This Research Topic “*Aquatic photosynthetic organisms under global change*” focuses on the direct and indirect effects of climate change on the life of microalga, macroalga, and aquatic angiosperms.

The increase in ocean temperature together with the increase of CO_2_ dissolution in the oceans leads to a change in the physico-chemical properties of seawater, including a decrease in pH, known as ocean acidification (Feely et al., 2009). Ocean acidification has pleiotropic effects on aquatic photosynthetic organisms because it promotes or decreases microalgal division rate (Gao et al., 2019), reduces the calcification of algae (Jin et al., 2017), increases the exposure of algae to UV radiation, and can negatively impact the symbiotic relationship between corals and microalgae (Fautin and Buddemeier, 2004), including the thermo-tolerant microalga *Symbiochlorum hainanensis* (Gong et al.). In this article Research Topic, Gong et al. reported on the impacts of elevated temperature and acidification alone or in combination on the biology of *S. hainanensis*. Overall, the data reveal that chloroplast adaptation constitutes one of the most important challenges of the adaptation of algae to climate change. Indeed, the contribution to this article Research Topic by Zhang et al. confirms and extends this conclusion to red macroalgae living in the intertidal zone, a very unique and challenging ecosystem because they are exposed, albeit temporarily, to extreme conditions such as fresh air, high light intensity, and UV radiation irradiation. High light exposure reduces photosynthesis and growth rate significantly. Moderate UV-A (315–400 nm) levels are beneficial for carbon fixation, nitrate uptake (Viñegla et al., 2006; Xu and Gao, 2010), and/or development of conchospores (Jiang et al., 2007) of some macroalgae but not all, including the red commercial macroalga genus *Pyropia* (Zhang et al., 2020) (formerly known as *Porphyra*; Sutherland et al., 2011). In this Research Topic, Zhang et al. also demonstrated that seawater acidification mitigates UV radiation on *Pyropia yezoensis* photosynthesis by modulating the synergy between photosystems. Mitochondrial metabolism is also important because it is involved in the control of the bloom of the dinoflagellate *Karenia mikimotoi* when nutrient availability and seawater acidity are altered (Liu et al.). Interestingly, it was found that nutrient limitations, especially phosphorous, can alleviate the negative impacts of acidification. Research in this field is particularly timely because these two factors are typical of global change (e.g., Gobler et al., 2017). Conversely, eutrophication can also increase the abundance of toxic microalgae (Anderson et al., 2002). The use of filter-feeding bivalve mollusks and submerged macrophytes can be an alternative to reduce indirectly the abundance of blooms. In this Research Topic, Du et al. presented a characterization of their impacts on phytoplankton bloom development by alleviating the eutrophication. In a nutrient-enriched freshwater mesocosm experiment, combining the filter-feeding bivalve *Cristaria plicata*, the cockscomb pearl mussel, and the macrophyte *Hydrilla verticillata* was highly efficient in decreasing the availability of nutrients, resulting in the suppression of bloom development, particularly by excluding cyanobacteria. While eutrophication can promote the occurrence of taxa, it can also jeopardize the survival of some of them such as the European aquatic plant *Luronium natans* (Alismataceae) (Makuch et al.).

In addition to aquatic photosynthetic organisms, submerged macrophytes occupy an important place in aquatic ecosystems, especially in shallow lakes and rivers (Hao et al., 2017), because they can maintain the physico-chemical properties and transparency of water (Wu et al., 2021). Like phytoplankton, submerged macrophytes also suffer from climate warming and eutrophication due to changes in abiotic variables alone or in combination with biotic variables (Hao et al., 2018; Matsuzaki et al., 2018). In their contribution, Wu et al. used mesocosms to determine the effects of climate warming and eutrophication on the growth of two aquatic plants, *Potamogeton crispus* and *Elodea canadensis*, at a seasonal scale (Zoppi et al. 2024). The latter taxon is recognized as an invasive species worldwide. The authors suggest that the variables explaining the variation in biomass are different for each season and that a synergetic effect of temperature and nutrients occurred rarely. At the annual scale, the overall results showed a direct positive effect of temperature rather than nutrient concentrations on *E. canadensis* biomass. Surprisingly, nutrient enrichment affected biomass by increasing competition among primary producers. Altogether, the study shows that ongoing climate warming and eutrophication will cause a transition in aquatic plant communities through selection effects.

Obviously, carbon metabolism is at the core of every reaction of photosynthetic organisms, in which the supply of inorganic carbon is of primary importance. This is particularly true for submerged organisms such as algae (e.g., Schoefs et al., 2017) but also for aquatic plants. In this Research Topic, two different cases are reported. The first one is the completely submerged marine land plant *Zoostera marina* (eelgrass) and the freshwater land plant *Ottela ovalifolia* with submerged and floating leaves. These plants differ not only in their autoecology but also in the source of inorganic carbon to which they have access. *Z. marina* only has access to HCO_3_^−^, whereas *O. ovalifolia* can fix either HCO_3_^−^ or CO_2_, depending on whether the leaves are submerged or emerged. *Z. marina* is a C_3_ plant, meaning that its photosynthetic capacity is limited by the activity of photorespiration, the efficiency of which decreases as the inorganic carbon concentration in the environment increases, a condition that drives global change. As explained previously, the CO_2_ accumulation in the ocean leads to its acidification, a process that, in turn, can impact photosynthesis. Using outdoor controlled *Z. marina* cultures, Celebi-Ergin et al. studied their responses to different inorganic carbon concentrations ranging from 55 to 2,121 µM. The data reveal a dynamic regulatory mechanism coupling i) energy capture capacity by pigments, ii) dissipation of the excess of absorbed energy through non-radiative energy dissipation mechanisms, typically non-photochemical quenching processes, and iii) photorespiration activity. Altogether, these three components of the photosynthetic machinery allow *Z. marina* to acclimate to the changing availability of inorganic carbon in the ocean. For their part, Liao et al. investigated how the freshwater aquatic plant *O. ovalifolia*, with two types of leaves uses CO_2_-concentrating mechanisms to optimize the uptake of inorganic carbon. At least two levels of adaptation were established. The first one concerns the morphological level with submerged leaves. Actually, submerged leaves are characterized by a larger specific surface area than floating leaves, so submerged leaves can better absorb dissolved inorganic carbon. The second level of adaptation relies on the involved carbon fixation cycle. In floating leaves, inorganic carbon is fixed directly on ribulose bisphosphate by ribulose-1,5-bisphosphate carboxylase oxygenase in the Calvin–Benson–Bassham cycle (C_3_ metabolism), whereas in submerged leaves, inorganic carbon is pre-fixed on phospho*enol*pyruvate in the Hatch and Slack cycle (C_4_ metabolism). This difference, together with an activation of the external carbonic anhydrases, allows an optimized supply of dissolved inorganic carbon to the submerged leaves.

Global change is leading to an additional shortage of various natural resources, including water and fertilizers, which have already become scarce due to the growing populations and shrinking arable lands. To slow down this shortage, recycling processes need to be introduced, especially for wastewater, including that from aquaculture, which is enriched with organic nutrients. Recycling wastewater to produce biomass is interesting in the context of a circular economy. The paper presented by Villanova et al. fits into this framework. Three microalgae were tested for their ability to remove nitrogen and phosphate from the recirculating marine aquaculture wastewater to produce high-quality biomass. As expected, wastewater supported high biomass production, and its enrichment with valuable compounds only occurred when the biomass was stressed (e.g., Sayanova et al., 2017). Interestingly, using this two-step process, the biomass of all tested strains was rich in proteins, polyunsaturated fatty acids (PUFAs), and carotenoids.

The contributions to this thematic Research Topic clearly confirm that global change is already affecting all living organisms, even the smallest ones. Despite the continuous accumulation of data, it is still difficult to determine exactly what will happen to each of them and how communities and ecosystems will change. Many aspects of these transformations remain to be studied, described, and, above all, understood. Multidisciplinary approaches are needed to achieve these goals. This information may also be of interest for the development of new biotechnological approaches and/or the improvement of current processes to make them more environmentally sustainable.
